# Continuous Non-Invasive Hemodynamic Monitoring in Cirrhotic Patients—Friend or Foe?

**DOI:** 10.3390/medicina61030536

**Published:** 2025-03-19

**Authors:** Mirela Crihan, Alexandru Leonard Alexa, Dan Valean, Daniela Ionescu

**Affiliations:** 1 1st Department of Anesthesia and Intensive Care, “Iuliu Haţieganu” University of Medicine and Pharmacy, 400347 Cluj-Napoca, Romania; crihan_mirela@yahoo.com (M.C.);; 2 Research Association in Anesthesia and Intensive Care (ACATI), 400394 Cluj-Napoca, Romania; 3 Department of Surgery, “Iuliu Haţieganu” University of Medicine and Pharmacy, 400347 Cluj-Napoca, Romania; valean.d92@gmail.com; 4 Outcome Research Consortium, Cleveland, OH 44195, USA

**Keywords:** cirrhosis, noradrenaline, intensive care, hemodynamic monitoring, vasoconstriction

## Abstract

*Background and Objectives*: Liver cirrhosis leads to significant hemodynamic changes, particularly portal hypertension and a hyperdynamic circulatory state. Traditional invasive methods for hemodynamic monitoring, while accurate, carry risks such as infection and hemorrhage in a patient predisposed to these conditions. This study evaluates the accuracy of non-invasive continuous hemodynamic monitoring compared to a minimally invasive method in patients with decompensated liver cirrhosis. *Materials and Materials and Methods*: The study enrolled 51 patients with decompensated liver cirrhosis requiring continuous hemodynamic monitoring in the ICU. Patients underwent simultaneous monitoring via the minimally invasive FloTrac system and continuous non-invasive ClearSight sensor over 24 h, with measurements registered at 6 h intervals. Hemodynamic parameters measured included cardiac output (CO), cardiac index (CI), stroke volume (SV), stroke volume variation (SVV), systemic vascular resistance (SVR), and mean arterial pressure (MAP). *Results*: Significant discrepancies were observed between the two monitoring methods for most parameters, particularly CO, CI, and MAP, at most time intervals. However, SVV measurements showed no significant differences, indicating similar efficacy in assessing fluid responsiveness between the devices. *Conclusions*: The ClearSight system, although a valuable non-invasive alternative, demonstrated lower accuracy compared to the FloTrac system for hemodynamic measurements in patients with decompensated liver cirrhosis. Its effectiveness in assessing fluid responsiveness, particularly by SVV, suggests it could play a role in the monitoring of these patients, especially when invasive techniques have increased risks.

## 1. Introduction

Liver cirrhosis induces profound hemodynamic alterations, primarily manifested by increased portal venous pressure (portal hypertension) due to fibrotic changes within the liver [1]. This results in a hyperdynamic circulatory state characterized by elevated cardiac output and decreased systemic vascular resistance and the portal level contributes to complications such as variceal hemorrhage and ascites [1,2,3]. These hemodynamic changes become more pronounced as cirrhosis and portal hypertension progress, and the severity of the hyperdynamic state is directly associated with the risk of developing complications such as variceal bleeding and ascites. Furthermore, the degree of hyperdynamic circulation is a significant predictor of patient outcomes, including overall survival in those with advanced liver disease [3,4].

In the management of decompensated cirrhosis, hemodynamic monitoring is an essential element. In recent years, there has been rapid development in continuous non-invasive hemodynamic monitoring technologies [5]. Patients with advanced cirrhosis often present with hyperdynamic circulation characterized by a decrease in systolic and diastolic blood pressure, and an increase in heart rate and cardiac output (CO) [5,6,7]. Even though they are often described as the gold standard, the invasive methods are relatively complex procedures (depending on the type of catheter) and have certain periprocedural risks like infection, local hemorrhage (in patients with coagulation disturbances), or thrombosis [6,7].

The ClearSight (Edwards Lifesciences— Irvine, CA, USA) system allows for continuous monitoring of hemodynamic parameters through a non-invasive finger cuff. Continuous data offered by the ClearSight system enables proactive optimization of perfusion through hemodynamic management [8]. This system uses a cuff applied on a finger of the patient and is a method of measuring arterial pressure and hemodynamic parameters (CO, SV, SVV, etc.) using a disposable pneumatic cuff [8] called the “volume clamping” method described by Jan Penaz [9]. After 8 h of continuous monitoring on the same finger, the cuff should be switched to another finger. The total measurement time shall not exceed 72 h of continuous monitoring [9,10].

In our study, we aimed to investigate if this monitoring is comparable to minimally invasive monitoring in decompensated cirrhotic patients, taking into consideration coagulation disturbances due to liver dysfunction with consecutive hemorrhage and the risk of catheter infection [8,9].

The main objective was to assess the accuracy and reliability of this continuous non-invasive method for measuring key hemodynamic parameters, such as CO, SV, SVV, SVR, CI, SVI, SVRI, and mean arterial pressure, in comparison to the minimally invasive method.

## 2. Materials and Methods

This study was approved by the Ethics Committee of the “Iuliu Haţieganu” University of Medicine and Pharmacy Cluj-Napoca (approval no. 94/20 June 2023) and by the Ethics Committee of the Regional Institute of Gastroenterology and Hepatology “Prof. Dr. Octavian Fodor” Cluj-Napoca (approval no. 1652/2 February 2024).

After written informed consent was obtained from each patient (or relatives in case of severe hepatic encephalopathy), the study enrolled 51 patients between January 2023 and September 2024 with decompensated liver cirrhosis admitted to the ICU who required continuous hemodynamic monitoring, vasoactive support, and mechanical ventilation. Data collection was handled in compliance with the Declaration of Helsinki.

Inclusion criteria for patients were a diagnosis of decompensated liver cirrhosis (bacterial or viral sepsis, hemorrhage) requiring continuous hemodynamic monitoring; aged 18–80 years; ASA risk II–III.

Exclusion criteria for this study included: refusal to participate; age < 18 years or > 80 years, patients with documented cardiac arrhythmias; patients with severe coagulopathies contraindicating arterial catheter placement during the study timeframe; and patients with severe peripheral vasoconstriction (determined via doppler ultrasound screening). Repeated compared measurements of hemodynamic parameters registered comparatively were taken at 6 h intervals during continuous patient monitoring for 24 h with minimally invasive techniques (FloTrac with HemoSphere monitor—Irvine, CA 92614, USA) and continuous monitoring of hemodynamic parameters via non-invasive techniques.

### 2.1. Minimally Invasive Monitoring

Eligible patients underwent insertion of an arterial catheter into the radial artery using the Seldinger technique. Catheterization was performed under local anesthesia, and the correct catheter position was confirmed by ultrasonography. The hemodynamic parameters (cardiac output [CO], cardiac index [CI], stroke volume [SV], stroke volume indexed [SVI], stroke volume indexed [SVI], stroke volume variable [SVV], systemic vascular resistance [SVR], systemic vascular resistance indexed [SVRI], and mean arterial pressure [MAP]) were measured continuously for 24 h and registered at 6 h intervals. The first measurement (T0) was done at 6 h after admission. This time interval was necessary for patient admission, catheter insertion, fluid administration, as well as noradrenaline infusion set-up.

### 2.2. Continuous Non-Invasive Monitoring

Continuous non-invasive hemodynamic monitoring was performed using a ClearSight sensor. This non-invasive technique preserves tissue integrity, offering advantages such as operational simplicity and reduced risk of catheter bleeding or infection. A disposable pneumatic cuff was applied to a finger (the middle finger) of each patient, continuously inflating and deflating in accordance with the volume clamping method previously discussed. The ClearSight system utilizes a self-calibration algorithm that periodically recalibrates the measurements using a cardiac reference system to account for the vertical displacement between the finger cuff and the heart [11,12]. Hemodynamic parameters were registered comparatively at 6 h time intervals for 24 h. Each study patient had a total of 4 comparative measurements of hemodynamic monitoring (T0, T1, T2, T4).

### 2.3. Statistics

Data collection was handled in compliance with EU General Data Protection Regulation legislation 2016/679 on the protection of individuals with regard to the processing of personal data.

The data were checked for normal distribution and comparisons of the mean values using *t*-tests for independent variables were performed. Based on the comparative cardiac output measurements of the first 5 patients, we calculated that a sample size of 44 patients would be necessary to attain a study power of 90%. We enrolled 51 patients to cover eventual dropouts.

The statistical analysis of the data was performed using IBM SPSS v26.0. Comparisons of the mean values between two variables were performed using a t-test. Comparisons of the mean between three or more variables were performed using ANOVA. A *p* < 0.05 was considered statistically significant.

## 3. Results

A final roster of 51 patients were enrolled in and completed the study. The demographic and clinical data of the study group are shown in Table 1.

The biochemical laboratory parameters and clinical score data are shown in Table 2.

The comparative time interval measurements (T0, T1, T2, T3) and hemodynamic values of the study group population (n = 51) are shown in Table 3.

**Figure 1:**
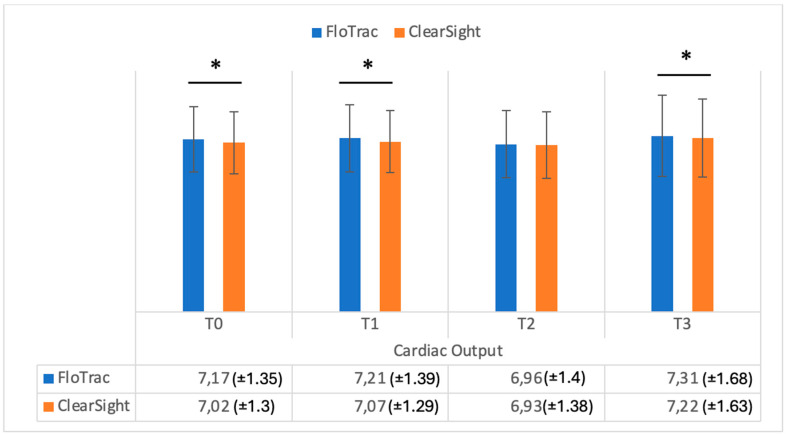
Cardiac output (liters/minute) measurements from FloTrac and ClearSight sensors. Data are represented as the mean values ± SD and the asterisks (*) indicate significant differences at *p* < 0.05.

**Figure 2:**
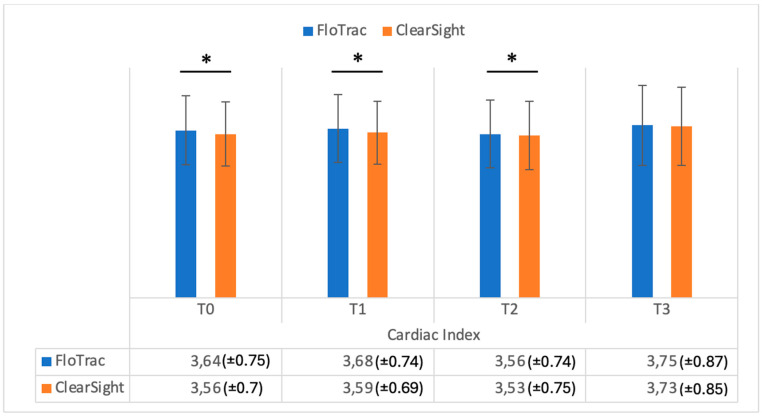
Cardiac index (liters/minute/square meter) measurements from FloTrac and ClearSight sensors. Data are represented as the mean values ± SD and the asterisks (*) indicate significant differences at *p* < 0.05.

**Figure 3:**
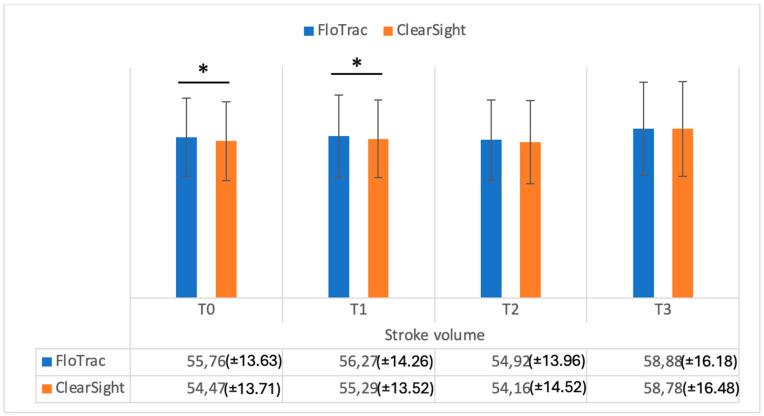
Stroke volume (mL/beat) measurements from FloTrac and ClearSight sensors. Data are represented as the mean values ± SD and the asterisks (*) indicate significant differences at *p* < 0.05.

**Figure 4:**
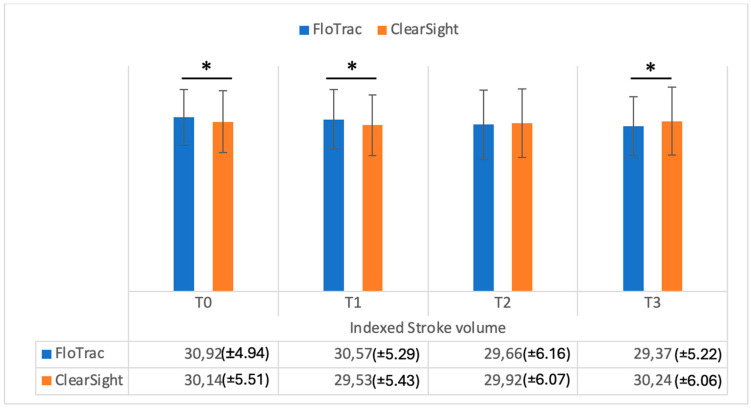
Indexed stroke volume (mL/beat/square meter) measurements from FloTrac and ClearSight sensors. Data are represented as the mean values ± SD and the asterisks (*) indicate significant differences at *p* < 0.05.

Figure 1 shows the data registered on cardiac output in the study groups, while Figure 2 shows the cardiac index in the study groups. As can be seen in Figure 1, cardiac output values were significantly different when measured comparatively (FloTrac vs. ClearSight — Irvine, CA, USA) at T0, T1, and T3 (*p* = 0.001, *p* = 0.002, *p* = 0.002). Cardiac index (CI) values were also significantly different between methods at T0, T1, and T2 (*p* = 0.001, *p* = 0.001, *p* = 0.03) (Figure 2). Regarding stroke volume (SV), as can be seen in Figure 3, significantly different values were registered at T0 and T1 (*p* = 0.001, *p* = 0.002), while indexed stroke volume (SVI) had significant differences at T0, T1, and T3, as seen in Figure 4 (*p* = 0.001, *p* = 0.001, *p* = 0.02).

**Figure 5:**
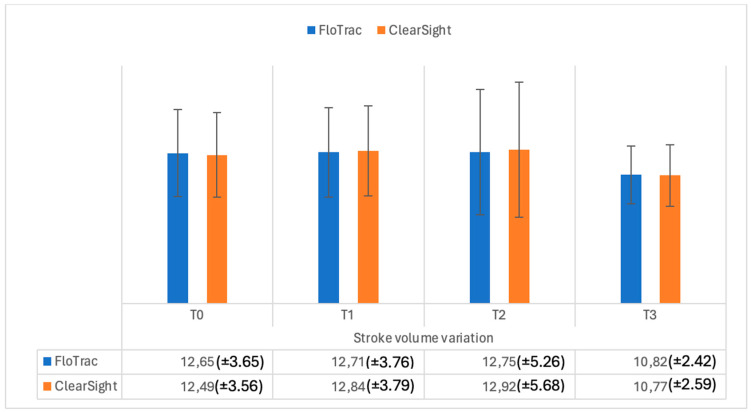
Stroke volume variation (%) measurements from FloTrac and ClearSight sensors. Data are represented as the mean values ± SD.

Stroke volume variation (SVV) did not differ at any time interval (Figure 5) between FloTrac and ClearSight — Irvine, CA, USA (*p* = 0.29; *p* = 0.41; *p* = 0.36; *p* = 0.69, respectively).

**Figure 6:**
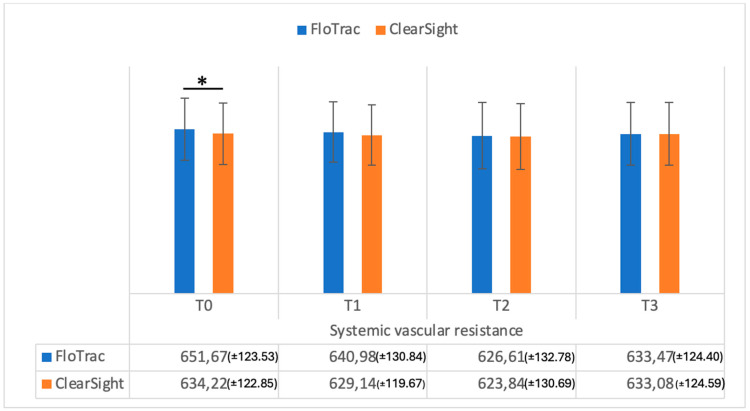
Systemic vascular resistance (dynes/cm^5^) measurements from FloTrac and ClearSight sensors. Data are represented as the mean values ± SD and the asterisk (*) indicates a significant difference at *p* < 0.05.

**Figure 7:**
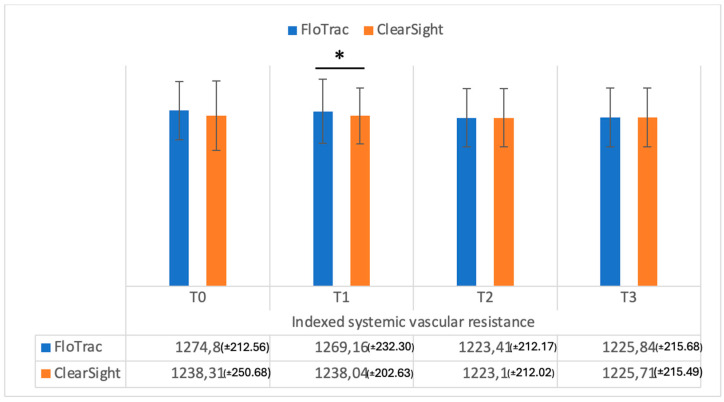
Indexed systemic vascular resistance (dynes/cm^5^/m^2^) measurements from FloTrac and ClearSight sensors. Data are represented as the mean values ± SD and the asterisk (*) indicates a significant difference at *p* < 0.05.

Systemic vascular resistance (SVR) values were significantly different (*p* = 0.001) between the methods at T0 (Figure 6), while indexed systemic vascular resistance (SVRI) was significantly different only at T1 (*p* = 0.01), as shown in Figure 7, and did not differ at the other time intervals.

**Figure 8:**
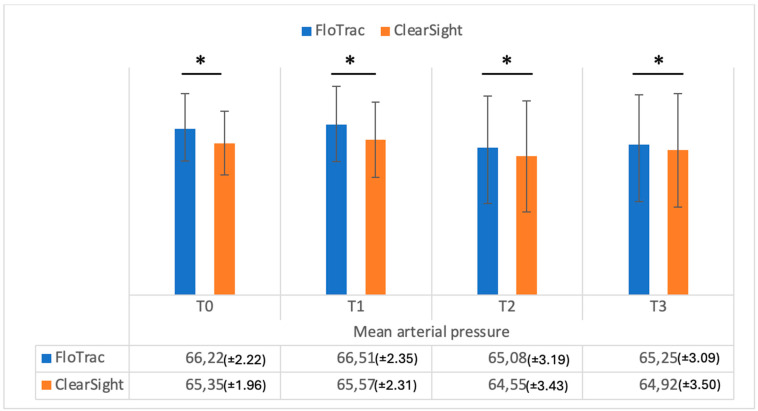
Mean arterial pressure (mmHg) measurements from FloTrac and ClearSight sensors. Data are represented as the mean values ± SD and the asterisks (*) indicate significant differences at *p* < 0.05.

Mean arterial pressure (MAP) values (FloTrac vs. ClearSight — Irvine, CA, USA) were significantly different at all intervals, as can be seen in Figure 8 (T0—*p* = 0.001; T1—*p* = 0.001; T2—*p* = 0.001; T3—*p* = 0.01, respectively). It can also be seen that MAP as measured by FloTrac had higher values as compared with ClearSight at any time interval.

**Figure 9:**
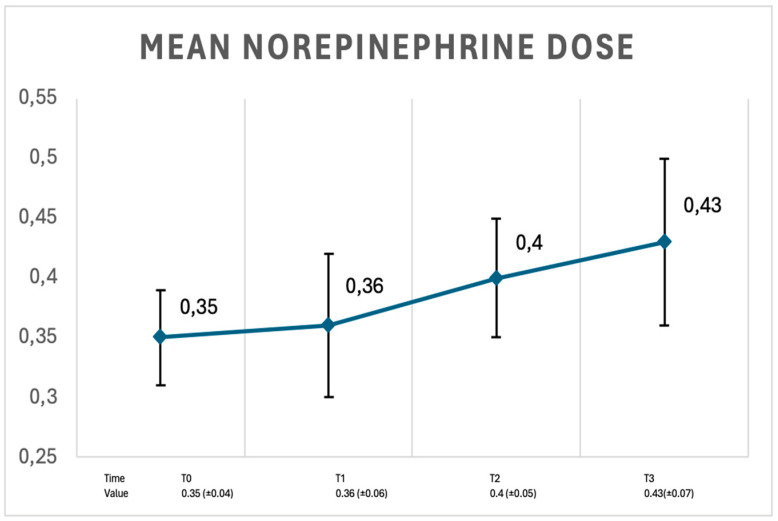
Mean norepinephrine dose (mcg/kg/min) in study group patients at study time intervals. Data are represented as the mean values ± SD.

The noradrenaline (norepinephrine) dose in the study group was recorded at every 6 h interval and varied, as shown in Figure 9.

## 4. Discussion

In our study, the hemodynamic parameters CO, CI, SV, SVV, SVI, SVR, SVRI, and MAP, were measured comparatively by two methods, minimally invasive and continuous non-invasive monitoring (FloTrac and ClearSight sensor), in decompensated cirrhotic patients. The ClearSight sensor measurements were statistically significantly different for most parameters, except for stroke volume variation (SVV), where there were no significant differences at any time interval. Most of discrepancies were registered at T0, T1, and T3.

Patients with decompensated liver cirrhosis have characteristic hemodynamic changes, such as systemic vasodilation and hyperdynamic circulation, initially increased cardiac output (CO) and cardiac index (CI), as well as decreased peripheral vascular resistance [13].

These hemodynamic characteristics may complicate hemodynamic measurements, as the FloTrac and ClearSight algorithms may be influenced by decreased blood volume and vascular tone [14]. For example, marked vasodilatation and decreased peripheral vascular resistance may lead to discrepancies in the comparative measurement of parameters such as SVR and MAP, especially in times of severe decompensation or after vasoactive drug administration [15].

There are relatively few studies on comparative measurements of hemodynamic parameters between minimally invasive and continuous non-invasive methods. Most of the studies were done perioperatively. Thus, Cho et al. found that ClearSight did not provide clinically acceptable interchangeability for cardiac output (CO) and systemic vascular resistance (SVR) compared to traditional invasive methods like pulmonary artery catheterization (PAC) during liver transplantation [7]. This was likely due to the hyperdynamic circulation and low SVR characteristic of cirrhotic patients, which made ClearSight less reliable, particularly during rapid hemodynamic changes caused by bleeding or drug administration [7]. However, in contrast with our results, for parameters like mean arterial pressure (MAP), ClearSight showed acceptable accuracy, making it potentially useful for certain non-invasive monitoring situations [7,16].

Hemodynamic assessment in patients with cirrhosis is essential for appropriate fluid and vasopressor management [17]. Measures such as cardiac output (CO), stroke volume (SV), and systemic vascular resistance (SVR) play a critical role in monitoring these patients, but non-invasive ClearSight technologies may have limitations in patients with marked vasodilation [18]. Sumiyoshi et al. highlighted the limitations of the ClearSight system in accurately measuring hemodynamic parameters, particularly in patients undergoing abdominal aortic aneurysm (AAA) surgery [19]. The primary objective of their study was to demonstrate the system’s inability to provide reliable cardiac index (CI) and systemic vascular resistance index (SVRI) measurements during significant hemodynamic changes induced by the surgical process, such as aortic clamping and declamping [19,20].

The ClearSight sensor is a widely used technology for hemodynamic monitoring, but its accuracy can be compromised under extreme conditions, such as hypotension with vasoactive medication or severe vasodilation, commonly seen in decompensated cirrhosis [21,22].

On the other hand, Lee’s study demonstrated that the non-invasive ClearSight method showed a statistically significant correlation with the traditional invasive monitoring method during single-lung thoracic surgery. Although systolic and diastolic blood pressures showed some discrepancies, they were close to acceptable levels compared to the invasive method [23].

There are several potential explanations for our findings compared to the studies mentioned above. Ascites and fluid retention are important characteristics in decompensated cirrhosis and may affect stroke volume (SV) and stroke volume index (SVI) measurements [24,25]. These factors may lead to overestimation or underestimation of the patient’s real hemodynamic status [24,25].

In our study, due to the fact that there were no significant differences in SVV measurements between FloTrac and ClearSight respectively, may indicate that the ClearSight sensor could be used to measure fluid responsiveness or fluid status with results comparable to the gold standard of invasive monitoring.

This may be attributed to several factors, including the patient’s volemic status and the characteristics of the calculation methods used by both technologies [26]. Patients with decompensated cirrhosis share similar pathophysiological characteristics with chronic heart failure [26]. Factors such as chronic vasodilation and mechanically controlled ventilation may also reduce normal SVV, making the measurements similar between devices [26,27]. In comparison to these results, Wang et al.’s study compared non-invasive ClearSight measurements with invasive Swan–Ganz catheter measurements in cardiac surgery patients and found that ClearSight provides accurate and reliable hemodynamic data, including stroke volume variation (SVV), comparable to traditional invasive methods [26].

The results of our study regarding fluid responsiveness monitoring are consistent with the established standards of good medical practice. The European Society of Intensive Care Medicine recommends the non-invasive option for hemodynamic monitoring and the initial assessment of the patient’s response to fluid therapy (fluid responsiveness) [27]. However, it may not be sufficient for the continuous evaluation of a critically ill patient [27]. For continuous, reliable monitoring and precise hemodynamic management, especially in cases where patients do not respond to fluid therapy and their hemodynamic instability deepens, minimally invasive techniques are recommended [28,29].

Recent data reinforces that the ClearSight sensor is an alternative, particularly in scenarios where invasive monitoring is not ideal or feasible [30,31]. In patients with decompensated cirrhosis, in which hemodynamic parameters are severely impaired, non-invasive monitoring can help to rapidly adjust vasopressors. However, in conditions where vasoconstriction induced by vasopressors affects the measurements, physicians should be cautious and supplement monitoring with additional invasive hemodynamic monitoring methods if the results are uncertain [21,26,30,31].

To our knowledge, this is the first study to evaluate two hemodynamic monitoring approaches: continuous non-invasive and invasive techniques. In cirrhotic patients, our main objective was to assess the accuracy and reliability of non-invasive methods in measuring key hemodynamic parameters, such as CO, SV, SVV, SVR, CI, SVI, SVRI, and mean arterial pressure, in comparison to invasive methods.

However, our study has some limitations. First is the small size of the study group, thus comparable with other similar studies published in the literature. We also did not corelate the magnitude of discrepancies in measurements with noradrenaline dose or the amount of fluids administered to study group patients.

## 5. Conclusions

Our study showed no significant difference in measuring SVV between continuous non-invasive and minimally invasive methods, while significant discrepancies were observed when comparing minimally invasive with non-invasive monitoring for cardiac output (CO), cardiac index (CI), and mean arterial pressure (MAP), in patients with decompensated cirrhosis. Further studies on large groups of patients are needed to quantify the role of continuous non-invasive methods.

## Figures and Tables

**Table 1:** The demographic data of the study group (*n* = 51). Data are represented as mean values ± SD or as numbers and percentages.

Age (years)	58.8 (±11.77)
ASA II/III (n, %)	22 (43.13)/29(56.86)
Gender F/M (n, %)	15 (29.4)/36(70.6)
Etiology of cirrhosisEthanolic/viral/autoimmune (n, %)	30 (58.8)/18(35.2)/3(5.8)

**Table 2:** The laboratory and clinical data of the study group. Data are represented as mean values ± SD or as numbers and percentages.

Total bilirubin (mg/dL)	10.86 (±12.07)
GOT/GPT (U/L)	850 (±1760)/427 (±764)
Cholinesterase (U/L)	2292 (±1033)
Fibrinogen (mg/dL)	202 (±111)
Albumin (g/dL)	2.96 (±0.39)
International normalized ratio (INR)	2.29 (±1.10)
Platelets (μL)	111,666 (±75,133)
SOFA score	13 (±5)
Apache II score	25 (±14)
MELD-Na score	22 (±11)
Child–Pugh A/B/C (*n*, %)	4 (7.84)/18 (35.28)/29 (56.84)
Acute kidney injury stage I/II/III (*AKI*) (*n*, %)	21 (41.16)/29 (56.84)/10 (19.6)
CLIF-C ACLF grade 1/2/3 (Acute-on-Chronic Liver Failure) (*n*, %)	7 (13.72)/13 (25.48)/31 (60.76)

**Table 3:** The statistical values of study group analyses comparing FloTrac and ClearSight hemodynamic measurement values at different time intervals. Data are represented as mean values ± SD.

Measurement	Parameter	FloTrac	ClearSight	*p*
T0	CO	7.17 (±1.35)	7.02 (±1.30)	0.001
	CI	3.64 (±0.75)	3.56 (±0.70)	0.001
	SV	55.76 (±13.63)	54.47 (±13.71)	0.001
	SVI	30.92 (±4.94)	30.14 (±5.51)	0.001
	SVV	12.65 (±3.65)	12.49 (±3.56)	0.29
	SVR	651.67 (±123.53)	634.22 (±122.85)	0.001
	SVRI	1274.8 (±212.5)	1238.31 (±250.68)	0.09
	MAP	66.22 (±2.20)	65.35 (±1.96)	0.001
T1	CO	7.21 (±1.39)	7.07 (±1.29)	0.002
	CI	3.68 (±0.74)	3.59 (±0.69)	0.001
	SV	56.27 (±14.26)	55.29 (±13.52)	0.002
	SVI	30.57 (±5.29)	29.53 (±5.43)	0.001
	SVV	12.71 (±3.76)	12.84 (±3.79)	0.41
	SVR	640.98 (±130.84)	629.14 (±119.67)	0.07
	SVRI	1269.16 (±232.30)	1238.04 (±202.63)	0.01
	MAP	66.51 (±2.35)	65.57 (±2.31)	0.001
T2	CO	6.96 (±1.40)	6.93 (±1.38)	0.1
	CI	3.56 (±0.74)	3.53 (±0.75)	0.03
	SV	54.92 (±13.96)	54.16 (±14.52)	0.13
	SVI	29.66 (±6.16)	29.92 (±6.07)	0.13
	SVV	12.75 (±5.26)	12.92 (±5.68)	0.36
	SVR	626.61 (±132.78)	623.84 (±130. 69)	0.26
	SVRI	1223.41 (±212.17)	1223.10 (±212.02)	0.37
	MAP	65.08 (±3.19)	64.55 (±3.43)	0.001
T3	CO	7.31 (±1.68)	7.22 (±1.63)	0.002
	CI	3.75 (±0.87)	3.73 (±0.85)	0.24
	SV	58.88 (±16.18)	58.78 (±16.48)	0.49
	SVI	29.37 (±5.22)	30.24 (±6.06)	0.02
	SVV	10.82 (±2.42)	10.77 (±2.59)	0.69
	SVR	633.47 (±124.40)	633.08 (±124.59)	0.25
	SVRI	1225.84 (±215.68)	1225.71 (±215.49)	0.55
	MAP	65.25 (±3.09)	64.92 (±3.50)	0.01

## Data Availability

The datasets used or analyzed during the current study are available from the corresponding author on request.

## Abbreviations

The following abbreviations are used in this manuscript:

AAA	abdominal aortic aneurysm
CO	cardiac output
CI	cardiac index
ICU	intensive care unit
SV	stroke volume
SVI	indexed stroke volume
SVV	stroke volume variation
SVR	systemic vascular resistance
SVRI	indexed systemic vascular resistance
MAP	mean arterial pressure
PAC	pulmonary artery catheterization
